# The lognormal distribution as a fit to symptom durations in the range 0-2 years for 26,000 cancer patients.

**DOI:** 10.1038/bjc.1987.267

**Published:** 1987-11

**Authors:** R. F. Mould, I. W. Hanham, B. F. McSweeney, D. R. Myles

**Affiliations:** Department of Medical Physics, Westminster Hospital, London, UK.


Br. J. Cancer (1987), 56, 687–689 © The Macmillan Press Ltd., 1987

SHORT COMMUNICATION

### The lognormal distribution as a fit to symptom durations in the range 0–2 Years for 26,000 cancer patients

R.F. Mould[1], I.W.F. Hanham[2], B.F.D. McSweeney[1] & D.R. Myles[1]

[1]*Department of Medical Physics; and* [2]*Department of Radiotherapy and Oncology, Westminster Hospital, London SW1P 2AP, UK.*

Very little information on symptom durations of cancer patients has been published in the literature and not even a table of typical symptom duration ranges for different cancer sites can be found. Nevertheless data on symptom duration if available can provide useful information when studying the natural history of the disease and when investigating possible correlations between symptom duration and post-treatment survival (Mould, 1985). However, when cancer registries changed from manual to computer systems, many which previously stored symptom durations discontinued this practice. In addition, during system changeovers some registries actually destroyed the relevant record cards from the earlier registration years. These factors make it extremely difficult in the United Kingdom to now obtain raw data on symptom duration. Consequently we have been fortunate in obtaining some 29,000 symptom durations for 26 different cancer sites. In a few instances the numbers were sufficiently large and the data sufficiently detailed for us to study site subgroups. These few subgroups were:

By stage (1, 2, 3, 4) for breast and cervix;
By anatomical subsite (glottic, supraglottic, subglottic) for larynx;
By treated or untreated group for rectum.

There were insufficient numbers of cases for time trends to be studied. The records were obtained from eight cancer registries in London, Birmingham, Bristol, Liverpool, Manchester and Sutton (Table I).

One disadvantage of recorded symptom durations is that patients often round their estimates to figures such as 3, 6, 12 or 24 months. However, when there was a relatively large number in a particular site group we repeatedly grouped the frequency data for model fitting using different symptom duration intervals. It was found that this did not alter the final outcome and therefore rounding errors do not significantly bias our data. Using a chi-squared test, a significance level of $P>0.05$ was chosen to accept the hypothesis that the observed symptom durations in the range 0–2 years are a good fit to a lognormal model for 0–2 years. It is emphasised that our technique was to estimate the lognormal parameters by fitting the model to the data and that the technique was not to fix a given pair of parameters $M$ and $S$ and then in effect say 'Does this fit, $P>0.05$?'. It should therefore be realised that it is not valid to use the model to predict symptom duration patterns beyond 2 years. Our decision to truncate the observed data at 2 years was made to improve data validity, since we believe that any patient estimation of symptom duration in excess of 2 years could be prone to large errors. This view was supported when asking colleagues to estimate time lapses from events which occurred more than two years ago.

The lognormal distribution was chosen for study since it is known to represent many positively skewed frequency distributions in nature, such as infant mortality rates (Schrek & Lipson, 1941), response times for different drugs (Gaddum, 1945; Lea, 1945) and the survival times of cancer patients who die with their disease present (Boag, 1949), especially those with cancers of the cervix (Mould & Boag, 1975) and

**Table I** Grouping by registry, site and treatment period

| Registry code | Treatment period | Site group | Number of cases in group: From 0–2 years | All durations |
|---|---|---|---|---|
| WEST | 1945–70 | Bladder | 421 | 477 |
| BIRM | 1970–76 | Brain | 1,594 | 1,735 |
| MANC | 1962–74 | Breast, stage I | 290 | 313 |
| MANC | 1962–74 | Breast, stage II | 202 | 213 |
| MANC | 1962–74 | Breast, stage III | 587 | 740 |
| MANC | 1962–74 | Breast, stage IV | 389 | 522 |
| MANC | 1947–61 | Cervix, stage I | 525 | 561 |
| MANC | 1947–51 | Cervix, stage II | 678 | 712 |
| MANC | 1947–51 | Cervix, stage III | 580 | 614 |
| MANC | 1947–61 | Cervix, stage IV | 478 | 520 |
| GORD | 1947–76 | Colon | 531 | 569 |
| BIRM | 1970–76 | Hodgkin's disease | 732 | 764 |
| BIRM | 1970–76 | Kidney | 869 | 905 |
| LEDE | 1933–73 | Larynx, glottic | 1,418 | 1,572 |
| LEDE | 1933–73 | Larynx, subglottic | 152 | 172 |
| LEDE | 1933–73 | Larynx, supraglottic | 441 | 479 |
| LIVE | 1970–79 | Leukaemia | 441 | 447 |
| BRIS | 1955–67 | Lip | 423 | 492 |
| WEST | 1960–70 | Lung | 554 | 563 |
| BRIS | 1955–67 | Melanoma | 314 | 428 |
| LEDE | 1933–73 | Nasopharynx | 529 | 552 |
| BIRM | 1970–76 | Oesophagus | 3,519 | 3,609 |
| BRIS | 1955–68 | Oropharynx | 104 | 104 |
| THAM | 1975–77 | Ovary | 1,687 | 1,704 |
| THAM | 1975–77 | Pancreas | 2,136 | 2,162 |
| BRIS[a] | 1945–67 | Penis | 154 | 187 |
| THAM | 1975 | Prostate | 819 | 866 |
| GORD | 1947–76 | Rectum, treated | 864 | 921 |
| GORD | 1947–76 | Rectum, untreated | 99 | 109 |
| BRIS | 1955–60 | Skin, basal cell | 1,009 | 1,833 |
| BRIS | 1955–60 | Skin, squamous cell | 467 | 642 |
| WEST | 1945–70 | Stomach | 715 | 783 |
| BIRM | 1970–76 | Testis | 442 | 467 |
| LIVE | 1970–79 | Thyroid | 203 | 217 |
| LEDE | 1933–73 | Tongue | 516 | 548 |
| LIVE | 1970–79 | Uterus | 1,067 | 1,096 |
| LIVE | 1970–79 | Vagina | 70 | 71 |
| | | Totals | 26,019 (=91%) | 28,669 (=100%)[b] |

*Registry codes:* BIRM = Birmingham (West Midlands) Regional; BRIS = South West (Bristol + Plymouth) Regional; BRIS[a] = Plymouth only; GORD = Gordon Hospital, London; LEDE = Personal registry records of Dr M. Lederman, Royal Marsden Hospital, London; LIVE = Mersey (Liverpool) Regional; MANC = Christie Hospital, Manchester; THAM = Thames Region, Sutton; WEST = Westminster Hospital.

*Abbreviations:* Squamous = Squamous cell carcinoma; Basal = Basal cell carcinoma.

[b]*Note:* Only 9% of all recorded symptom durations were greater than 2 years and therefore have not been used in this analysis.

Correspondence: R.F. Mould.
Received 10 May 1987; and in revised form, 22 July 1987.

cancers of the head and neck (Mould *et al.*, 1976). The lognormal distribution is:

$$f(T)=\frac{1}{TS\sqrt{2\pi}}\exp(-[\log_e(T/M)]^2/2S^2).$$

Where $T$ is the symptom time

Where $M=\exp(\bar{x})$ and is median of the lognormal dist

Where $S$ is the standard deviation

$$S^2=\frac{1}{n-1}\sum_{i=1}^{n}[\log_e(x_i-\bar{x})]^2$$

and

$$\bar{x}=(1/n)\sum_{i=1}^{n}\log x_i$$

the $x_i$ are the individual symptom times.

Model fitting using chi-squared tests were accomplished using software written for use with the Hewlett-Packard 86B microprocessor based Westminster Hospital Cancer Registry (Mould, 1982) and the results are shown in Table II for 37 data groups relating to 26 cancer sites. An alternative model fitting computer program is published by McKintosh & McKintosh (1980) in an appendix to their book on modelling in endocrinology.

The two parameter lognormal model provided a good fit, $P>0.05$, for all 37 data groups although the values of $M$ and $S$ differed among the groups. Our estimates of the minimum chi-squared parameters, termed $M^*$ and $S^*$, are given in Table II together with information on whether a fit to the model was obtained with different values of $S$ in the range 0.30 to 0.90, with discrete intervals of 0.05.

The minimum chi-squared estimate of the mean logtime, $M^*$ in Table II, is in good agreement with the observed median symptom duration, as would be expected. The range of $M^*$ for 35 of the 37 site groups is 9 months, $1\leq M^*<10$, with only basal cell carcinoma of the skin and stage IV breast cancer having larger values of $M^*$, respectively 18.8 and 12.5 months. Patients with late stage (III and IV) breast cancer with $M^*=9.9$ and 12.5 months in general delay longer before diagnosis than late stage cervical cancer patients for whom $M^*=5.0$ and 6.2 months. Although for early stage (I and II) breast cancer $M^*=1.3$ and 1.4 months, which is less than the

**Table II** Results of chi-squared goodness-of-fit lognormal model testing

| *Site group* | *Median of all durations (months)* | *Lognormal minimum chi-squared parameters* $M^*$ | $S^*$ | *M-values for different values of the lognormal parameter S for which a good fit was obtained, P>0.05. If a good fit was not obtained no value for M has been included in the table* $S=$ 0.30 | 0.35 | 0.40 | 0.45 | 0.50 | 0.55 | 0.60 | 0.65 | 0.70 | 0.75 | 0.80 | 0.85 | 0.90 |
|---|---|---|---|---|---|---|---|---|---|---|---|---|---|---|---|---|
| Bladder | 4.9 | 4.5 | 0.58 | — | — | — | — | 3.9 | 4.3 | 4.6 | 5.1 | — | — | — | — | — |
| Brain | 2.0 | 1.9 | 0.70 | — | — | — | — | — | — | 1.9 | 1.9 | 1.9 | 1.9 | — | — | — |
| Breast, stage I | 1.7 | 1.3 | 0.77 | — | — | — | — | — | — | 1.3 | 1.3 | 1.3 | 1.3 | 1.3 | 1.3 | — |
| Breast, stage II | 1.7 | 1.4 | 0.69 | — | — | — | — | — | 1.5 | 1.4 | 1.4 | 1.4 | 1.4 | 1.4 | 1.4 | — |
| Breast, stage III | 8.1 | 9.9 | 0.79 | — | — | — | — | — | — | — | — | 8.0 | 8.8 | 10.1 | 11.1 | — |
| Breast, stage IV | 12.9 | 12.5 | 0.70 | — | — | — | — | — | — | 11.1 | 11.1 | 12.5 | 14.0 | — | — | — |
| Cervix, stage I | 4.5 | 4.0 | 0.50 | — | — | — | 3.8 | 4.0 | 4.3 | — | — | — | — | — | — | — |
| Cervix, stage II | 3.8 | 4.0 | 0.52 | — | — | — | — | 3.9 | 4.2 | 4.5 | — | — | — | — | — | — |
| Cervix, stage III | 5.1 | 5.0 | 0.41 | — | — | 5.0 | 5.3 | — | — | — | — | — | — | — | — | — |
| Cervix, stage IV | 5.8 | 6.2 | 0.48 | — | — | — | 5.9 | 6.3 | 6.9 | — | — | — | — | — | — | — |
| Colon | 5.1 | 5.3 | 0.63 | — | — | — | — | — | — | 5.2 | 5.8 | 6.0 | 6.3 | — | — | — |
| Hodgkin's disease | 2.6 | 2.4 | 0.44 | — | — | — | 2.4 | — | — | — | — | — | — | — | — | — |
| Kidney | 1.9 | 1.7 | 0.62 | — | — | — | — | — | 1.7 | 1.7 | 1.7 | 1.7 | — | — | — | — |
| Larynx, glottic | 5.5 | 5.3 | 0.40 | — | — | 5.2 | — | — | — | — | — | — | — | — | — | — |
| Larynx, subglottic | 6.9 | 7.2 | 0.55 | — | — | — | — | 6.6 | 7.2 | 8.1 | 8.9 | — | — | — | — | — |
| Larynx, supraglottic | 5.0 | 5.1 | 0.53 | — | — | — | 4.8 | 5.0 | 5.4 | 5.8 | 6.2 | — | — | — | — | — |
| Leukaemia | 1.8 | 1.8 | 0.49 | — | — | — | 1.8 | 1.8 | 1.8 | — | — | — | — | — | — | — |
| Lip | 5.4 | 5.1 | 0.46 | — | — | — | 5.0 | 5.4 | — | — | — | — | — | — | — | — |
| Lung | 1.9 | 1.9 | 0.55 | — | — | — | 1.8 | 1.9 | 1.9 | 1.9 | 2.0 | — | — | — | — | — |
| Melanoma | 7.2 | 8.2 | 0.57 | — | — | — | — | 6.7 | 7.8 | 9.0 | — | — | — | — | — | — |
| Nasopharynx | 5.1 | 5.0 | 0.41 | — | — | 4.8 | 5.2 | 5.6 | — | — | — | — | — | — | — | — |
| Oesophagus | 2.4 | 2.4 | 0.38 | — | — | 2.5 | 2.8 | — | — | — | — | — | — | — | — | — |
| Oropharynx | 1.9 | 2.1 | 0.46 | — | — | 2.1 | 2.1 | 2.1 | 2.1 | 2.2 | — | — | — | — | — | — |
| Ovary | 1.7 | 1.6 | 0.58 | — | — | — | — | — | 1.6 | 1.6 | 1.6 | — | — | — | — | — |
| Pancreas | 0.9 | 1.0 | 0.64 | — | — | — | — | — | — | 1.1 | 1.0 | 1.0 | — | — | — | — |
| Penis | 5.8 | 5.6 | 0.65 | — | — | — | — | 5.6 | 5.6 | 5.6 | 5.6 | 5.6 | 5.6 | 5.6 | 5.8 | 6.2 |
| Prostate | 2.6 | 2.5 | 0.67 | — | — | — | — | — | 2.4 | 2.4 | 2.5 | 2.5 | 2.6 | 2.7 | 2.8 | — |
| Rectum, treated | 5.4 | 5.7 | 0.52 | — | — | — | — | 5.6 | 6.1 | — | — | — | — | — | — | — |
| Rectum, untreated | 6.6 | 7.2 | 0.56 | — | — | — | 6.7 | 6.8 | 6.8 | 7.5 | 7.9 | 8.1 | 8.3 | — | — | — |
| Skin, basal cell | 21.8 | 18.8 | 0.62 | — | — | — | — | — | 10.6 | 17.6 | 21.1 | — | — | — | — | — |
| Skin, squamous cell | 10.6 | 9.8 | 0.66 | — | — | — | — | — | — | 8.7 | 9.6 | 10.7 | — | — | — | — |
| Stomach | 4.0 | 4.1 | 0.61 | — | — | — | — | — | 3.9 | 4.1 | 4.4 | 4.7 | — | — | — | — |
| Testis | 2.6 | 2.4 | 0.55 | — | — | — | — | 2.4 | 2.4 | 2.4 | 2.5 | — | — | — | — | — |
| Thyroid | 3.8 | 3.9 | 0.47 | — | — | — | 3.9 | 4.0 | 4.1 | — | — | — | — | — | — | — |
| Tongue | 3.6 | 3.5 | 0.46 | — | — | 3.5 | 3.5 | 3.5 | — | — | — | — | — | — | — | — |
| Uterus | 3.6 | 3.6 | 0.46 | — | — | — | 3.6 | 3.8 | — | — | — | — | — | — | — | — |
| Vagina | 2.6 | 2.5 | 0.39 | 2.8 | 2.8 | 2.8 | 2.8 | 3.0 | 3.1 | — | — | — | — | — | — | — |
| Total number of groups for which a good fit to the data was obtained, $P>0.05$ | | | | 1 | 1 | 7 | 16 | 20 | 22 | 22 | 19 | 13 | 9 | 5 | 5 | 1 |

*Abbreviations:* Squamous = Squamous cell carcinoma; Basal = Basal cell carcinoma; $M^*$ = Minimum chi-squared estimate of the $S^*$ mean logtime given in months as distinct from a logarithm of months.

$M^* = 4.0$ months for cancer of the cervix. The data are relevant particularly for those who are trying to achieve earlier diagnosis of these two cancers.

The lognormal model for the prediction of long-term survival rates using a value assumed for the parameter $S$ in the range $S = 0.35$, to 0.40 has been shown to be satisfactory for cancer of the cervix (Mould & Boag, 1975) although values in the range $S = 0.45$ to 0.50 are better for cancer of the head and neck (Mould *et al.*, 1976). These models refer to cancer follow-up after treatment, whereas the data under discussion in this paper relate to the time before treatment. In this case (Table II), the optimum values of $S$ are in the range 0.50 to 0.65, which represents a different family of lognormal curves than those describing survival after treatment.

For 30 of the 37 site groups the lognormal was found to be a good fit for a range of values of $S$ differing by at least 0.10, and in nine of these 30 groups, differing by at least 0.20 (Table II). Over this range of values of $S$ for a given site group the chi-squared estimate of $M$ usually remained reasonably stable. It is therefore suggested that the lognormal distribution can be used in practice to provide a first estimate of the frequency distribution of cancer symptom durations in the range 0–2 years, particularly since no alternative method exists.

We are most grateful to the many people from the cancer registries who facilitated our efforts to obtain raw data on symptom duration, or who in some instances provided computer outputs for us with the data grouped in monthly intervals. Without such help this study would have been impossible. In particular we are indebted to Mrs Joy Adams, Dr Val Blair, Mrs Sandra Gravestock, Mr A.R.D. Kilburn, the late Dr M. Lederman, Miss Gwen Redmayne, Mr R. Skeet, Dr J.A.H. Waterhouse and Mrs Pat Watts.

**References**

BOAG, J.W. (1949). Maximum likelihood estimates of the proportion of patients cured by cancer therapy. *J.R. statist. Soc.* (Series B), **11**, 15.

GADDUM, J.H. (1945). Lognormal distributions, *Nature*, **156**, 463.

LEA, D.E. (1945). The biological assay of carcinogens. *Cancer Res.*, **5**, 633.

McKINTOSH, J.E.A. & McKINTOSH, R.P. (1980). *Mathematical Modelling and Computers in Endocrinology*. Springer-Verlag, Berlin.

MOULD, R.F. (1982). The Westminster Hospital Microprocessor Cancer Registry, *Br. J. Radiol.*, **55**, 897.

MOULD, R.F. (1985). Symptom duration and survival. *Br. J. Radiol.*, **58**, 1028.

MOULD, R.F. & BOAG, J.W. (1975). A test of several parametric statistical models for estimating success rate in the treatment of carcinoma cervix uteri. *Br. J. Cancer*, **32**, 529.

MOULD, R.F., HEARNDEN, T., PALMER, M. & WHITE, G.C. (1976). Distribution of survival times of 12,000 head and neck cancer patients who died with their disease. *Br. J. Cancer*, **34**, 180.

SCHREK, R. & LIPSON, H.I. (1941). Logarithmic frequency distributions. *Human Biology*, **13**, 1.


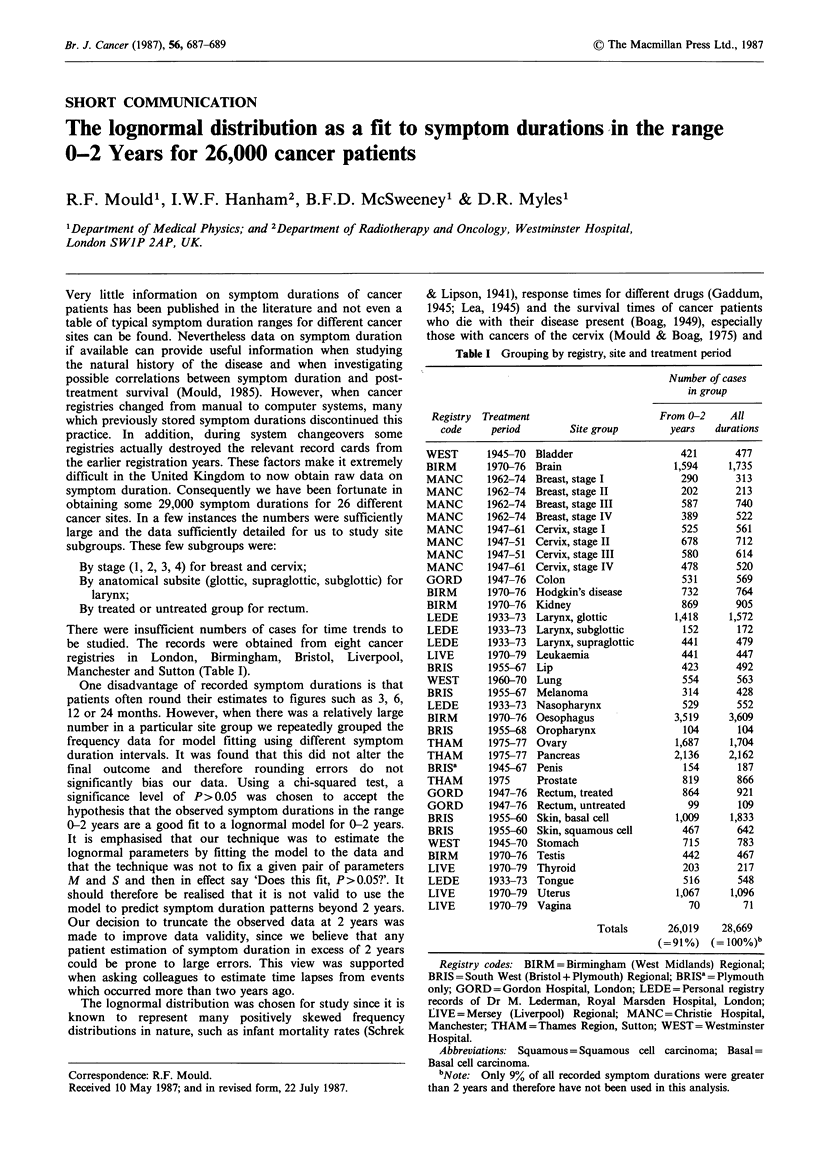

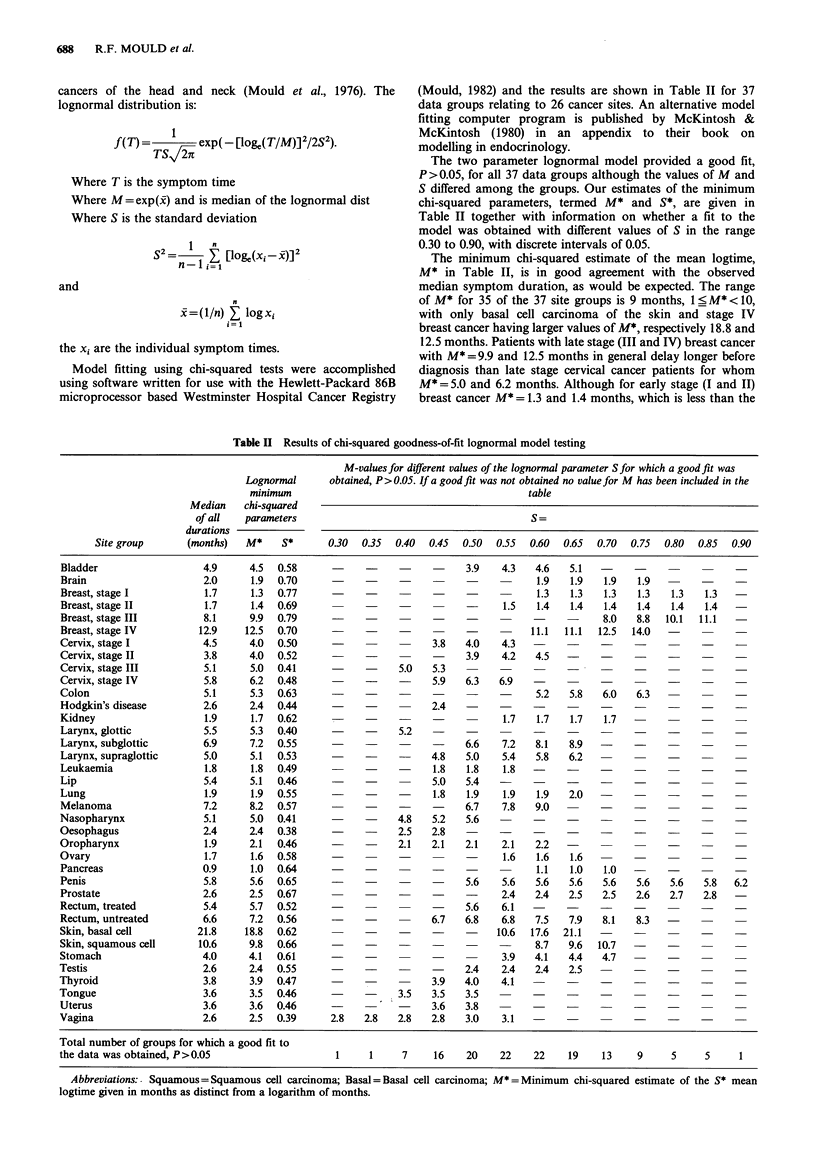

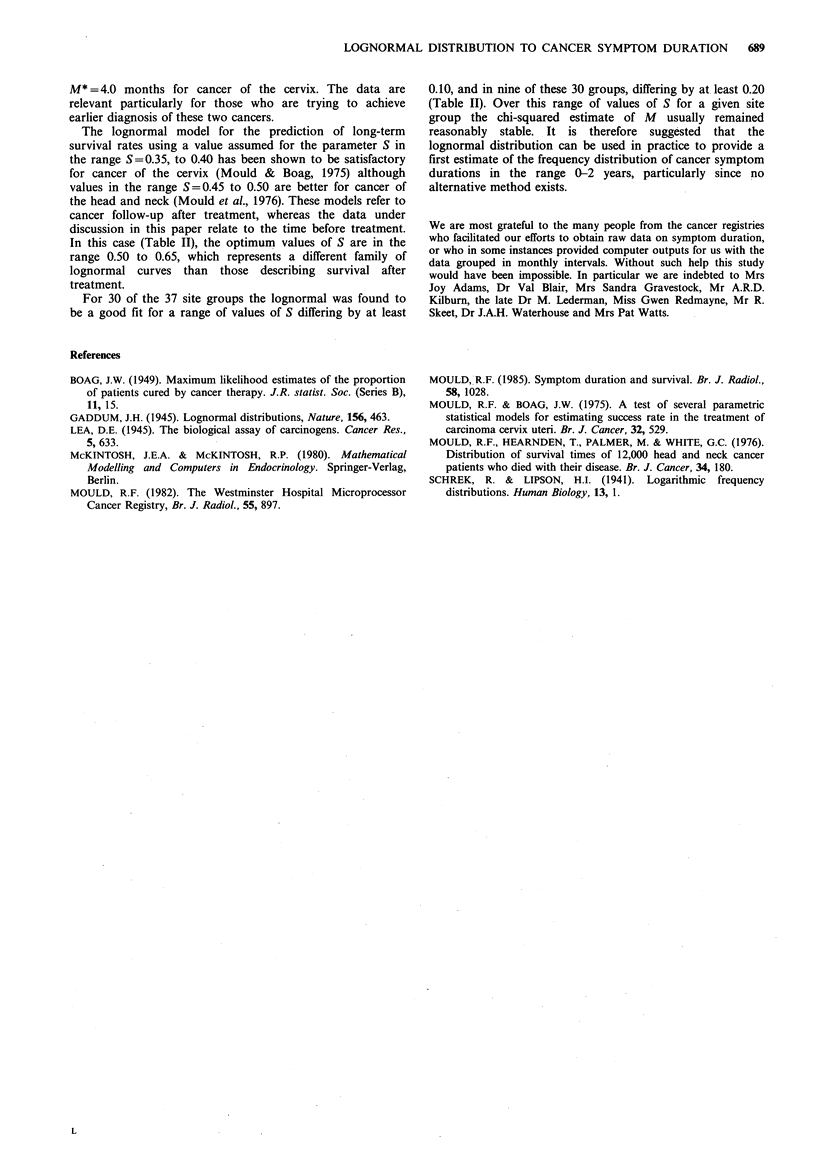


## References

[OCR_00353] Mould R. F., Boag J. W. (1975). A test of several parametic statistical models for estimating success rate in the treatment of carcinoma cervix uteri.. Br J Cancer.

[OCR_00358] Mould R. F., Hearnden T., Palmer M., White G. C. (1976). Distribution of survival times of 12,000 head and neck cancer patients who died with their disease.. Br J Cancer.

[OCR_00349] Mould R. F. (1985). Symptom duration and survival.. Br J Radiol.

[OCR_00345] Mould R. F. (1982). The Westminster hospital microprocessor cancer registry.. Br J Radiol.

